# Effects of Calcium and Signal Sensing Systems on *Azorhizobium caulinodans* Biofilm Formation and Host Colonization

**DOI:** 10.3389/fmicb.2020.563367

**Published:** 2020-09-16

**Authors:** Xiaolin Liu, Kaiye Zhang, Yanan Liu, Desheng Zou, Dandan Wang, Zhihong Xie

**Affiliations:** ^1^Key Laboratory of Coastal Environmental Processes and Ecological Remediation, Yantai Institute of Coastal Zone Research, Chinese Academy of Sciences, Yantai, China; ^2^College of Resources and Environment, University of Chinese Academy of Sciences, Beijing, China; ^3^National Engineering Laboratory for Efficient Utilization of Soil and Fertilizer Resources, College of Resources and Environment, Shandong Agricultural University, Tai’an, China; ^4^Center for Ocean Mega-Science, Chinese Academy of Sciences, Qingdao, China

**Keywords:** *Azorhizobium caulinodans*, calcium, biofilm, chemotaxis, quorum sensing, eDNA

## Abstract

Biofilm formation is important for establishing plants-microbe associations. The role of calcium on biofilm formation has been studied in many bacteria except rhizobia. In this study, we investigated the role of calcium for biofilm formation in *Azorhizobium caulindans*, which forms nodules in the stem and root of its host plant *Sesbania rostrata*. We found that calcium is essential for *A. caulindans* biofilm formation, in addition to the presence of extracellular matrix components, eDNA and proteins. Also, calcium-mediated biofilm formation was tested with chemotaxis, motility, cyclic di-GMP synthesis, and quorum sensing mutants. Finally, calcium was found to promote *S. rostrata* root colonization of *A. caulinodans*. In total, these results show that calcium is essential for *A. caulindans* biofilm formation, and it affects the interaction between *A. caulinodans* and host plant.

## Introduction

Environmental bacteria often form communities on biotic or abiotic surfaces called biofilms. Biofilm formation enables bacteria to survive in harsh environments or build an association with hosts (Tallawi et al., 2017). Biofilm formation can be beneficial to bacteria for several reasons: (1) Biofilm formation can protect bacteria under different stress conditions (Jefferson, 2004), (2) Biofilm formation can promote colonization of many bacteria and can allow bacteria to remain in a nutrient-rich niche (Jefferson, 2004), (3) Biofilm is a stage for the cooperation and communication between bacteria, and it is beneficial for bacteria to resist harsh environment together (Jefferson, 2004), and (4) Biofilm formation can generate niches that provide unique growth advantages. For example, biofilm formation enables free-living nitrogen-fixing rhizobacteria to fix nitrogen under aerobic conditions (Wang et al., 2017).

In biofilms, bacteria are coated with hydrated extracellular polymeric substances, including polysaccharides, proteins, nucleic acids, and lipids (Flemming and Wingender, 2010). The extracellular matrix provides a three-dimensional frame to stabilize the structure of biofilm, regulates the adhesion of bacteria to biotic or abiotic surface, and accommodates extracellular enzymes to digest biopolymers (Flemming and Wingender, 2010). Bacteria generate the extracellular matrix, and the components of the matrix changes depending on the bacterial species and growth conditions (Flemming and Wingender, 2010; Tallawi et al., 2017). In recent years, more and more extracellular matrix components from different bacteria have been characterized. Based on the extracellular matrix components, biofilm can be classified into two major classes: polysaccharides-predominant or polypeptides-predominant (Tallawi et al., 2017). In addition to polysaccharides and proteins, many bacteria also contain additional components, such as extracellular DNA (eDNA) and fimbriae in biofilm (Tallawi et al., 2017).

The development of both major classes of biofilm, represented by *Pseudomonas aeruginosa* (Ma et al., 2009) and *Staphylococcus aureus* (Hobley et al., 2015), occurs in similar sequential processes, including the initiation of biofilm formation, adhesion and maturation, and biofilm dispersal (Vlamakis et al., 2013). At the initial stage of biofilm formation, flagella enable bacteria to move in a relatively long-range surface and further increase the likelihood of approaching and adhering to surface (Karatan and Watnick, 2009; Belas, 2014). The flagellar mechanosensing of surfaces switches bacteria from a motile to a sessile lifestyle, inducing biofilm formation (Belas, 2014). The initial adherence is reversible, but once bacterial cells adhere to a surface irreversibly, the function of flagella and flagellar synthesis genes is inhibited (Zamorano-Sánchez et al., 2019). During the adhesion and maturation stages of biofilm formation, a signaling molecule, cyclic dimeric guanosine monophosphate (c-di-GMP) is involved in the regulation of motility and biofilm formation (Yang et al., 2018). The concentration of intracellular c-di-GMP correlates with the transition from a motile to a biofilm lifestyle. For example, in *P. aeruginosa*, high c-di-GMP levels inhibit motility and enhance biofilm formation (Simm et al., 2004). Diguanylate cyclases (DGCs) catalyze a reaction that generates c-di-GMP from two molecules of GTP. In contrast, phosphodiesterases (PDEs) catalyze the breakdown reaction of c-di-GMP. The detachment and dispersal of cells from biofilm occurs when the size of biofilm grows to a cell density threshold where cells no longer have access to nutrients (Karatan and Watnick, 2009). Cell-cell communication is also involved in biofilm formation. Quorum sensing is a density-dependent system of cell-cell communication by the release of chemical signals called autoinducers (Mukherjee and Bassler, 2019). For example, the biofilm of *Vibrio cholerae* is promoted at low cell density (Hammer and Bassler, 2003), while quorum sensing promotes the biofilm of *Pseudomonas aeruginosa* at high cell density (Davies et al., 1998).

The biofilm formation is also affected by many nutritional and environmental conditions. For some bacteria, such as *Sinorhizobium meliloti* (Rinaudi et al., 2006) and *Dickeya zeae* (Huang et al., 2019), nutrient sources enhance biofilm formation, while extreme conditions such as extreme temperature or pH prevents biofilm formation. But for others, such as in *Azorhizobium caulinodans*, lack of nutrient sources increases biofilm formation (Jiang et al., 2016; Liu et al., 2018b). In addition to nutrient and physical factors like pH and temperature, biofilm formation also can be affected by multivalent cations, including Ca^2+^, Cu^2+^, Mg^2+^, and Fe^3+^. Calcium (Ca^2+^) can affect biofilm formation positively or negatively in many bacteria, such as *P. aeruginosa* (Sarkisova et al., 2005) and *Vibrio cholerae* (Bilecen and Yildiz, 2009), using diverse mechanisms. For example, calcium can cause extensive formation of ordered helices of polysaccharides (Sutherland, 2001) or function as a small-molecule signal (Tischler et al., 2018).

*Azorhizobium caulinodans* is a symbiont of a tropical legume plant *Sesbania rostrata* (Dreyfus et al., 1988). *A. caulinodans* cannot only fix atmospheric nitrogen with *S. rostrata* in a symbiotic state (Dreyfus and Dommergues, 1981), but also can fix nitrogen in a free-living state (Dreyfus et al., 1983) or in association with non-legume plants, such as rice and wheat (Liu et al., 2017a). During the establishment of rhizobia-plant associations, biofilm formation on plant surfaces plays an important role (Yaryura et al., 2008; Xu et al., 2018). However, the process of biofilm formation of *A. caulinodans* and the role of biofilm formation on its early symbiosis with *S. rostrata* are less studied.

In this study, we used tryptone and yeast extract (TY) rich medium instead of L3 minimal medium to form biofilms, and found calcium is an essential factor for *A. caulinodans* biofilm formation. We not only determined the Ca^2+^-mediated biofilm of *A. caulinodans* is an eDNA predominant type, but also determined the roles of chemotaxis, motility, c-di-GMP, and quorum sensing on different stages of Ca^2+^-mediated biofilm formation and development. Finally, the positive effect of Ca^2 +^ on the establishment of *A. caulinodans*- *S. rostrata* associations was also characterized.

## Results

### Altering *A. caulinodans* Biofilm Growth Condition Reveals a Crucial Role for Calcium

**FIGURE 1:**
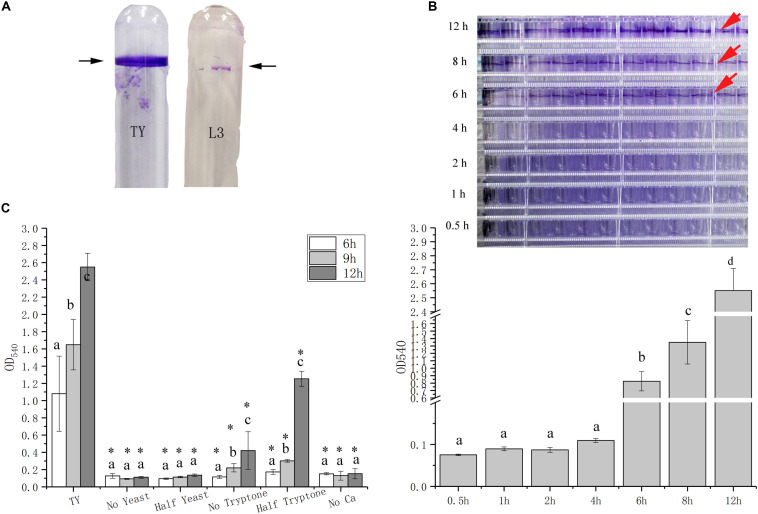
Biofilm formation of *Azorhizobium caulinodans* at various timepoints with different media components. **(A)** Biofilm formation of *A. caulinodans* with TY or L3 medium. Representative images of *A. caulinodans* biofilm on the wall of glass tubes after culturing for 5 days. **(B)** *A. caulinodans* biofilm formation in TY medium from 0.5 to 12 h. Images on the top are representative biofilms on the wall of 96-well plates. **(C)** Biofilm formation of *A. caulinodans* after 6, 9, and 12 h, when different components were removed from TY medium. Values are shown as the means and standard deviations from at least three independent experiments. The same letter above the error bars indicates not statistically different with results after culturing for 0.5 h **(B)** or under same conditions **(C)** by a Duncan’s test. Asterisks (^∗^) means *P* < 0.05 vs. the results after same time with TY medium by a Student’s *t* test.

Previously, we used the L3 minimal medium, which uses 10 mM sodium lactate and 10 mM ammonium chloride as sole carbon and nitrogen sources, to explore the biofilm formation of *A. caulinodans* (Liu et al., 2018a). It is easy to remove ammonium chloride from L3 medium and construct a nitrogen free environment for *A. caulinodans* to fix nitrogen, thus L3 minimal medium is better to study the physiological variance of *A. caulinodans* under nitrogen fixation and nitrogen-replete conditions. *A. caulinodans*, however, can only form a weak biofilm when grown in L3 medium for 3–5 days. Interestingly, we found that *A. caulinodans* forms a thicker and broader biofilm in TY medium compared to L3 medium after 3–5 days (Figure 1A). Because *A. caulinodans* cells grow better in L3 medium than in TY medium (Supplementary Figure S1), we suspected that there might be potential signals in TY medium promoting the biofilm formation of *A. caulinodans*.

To better investigate the biofilm formation of *A. caulinodans* with TY medium, we first tried to determine the kinetics of biofilm formation. The biofilm biomass of *A. caulinodans* was assessed by crystal violet (CV) staining assay over 12 h. Although earlier biofilm experiments assessed at biofilm formation after 3–5 days in L3 media, *A. caulinodans* can form biofilm after 6 h in TY media (Figure 1B). Thus, all the work of this study was performed in TY medium within 6–12 h. To determine if specific signals within TY media promote biofilm formation, we tested the ability of *A. caulinodans* to form biofilm in TY media that lack either some or all portions of tryptone, yeast extract, and calcium. The results showed that biofilm formation is dependent on calcium and yeast extract (Figure 1C). Because the absence of yeast extract in TY media significantly affected the growth of *A. caulinodans* (Supplementary Figure S2), we only focused on the role of calcium on biofilm formation in further work.

### Determining How Various Concentrations of Calcium Alter Characteristics of *A. caulinodans* Biofilm

**FIGURE 2:**
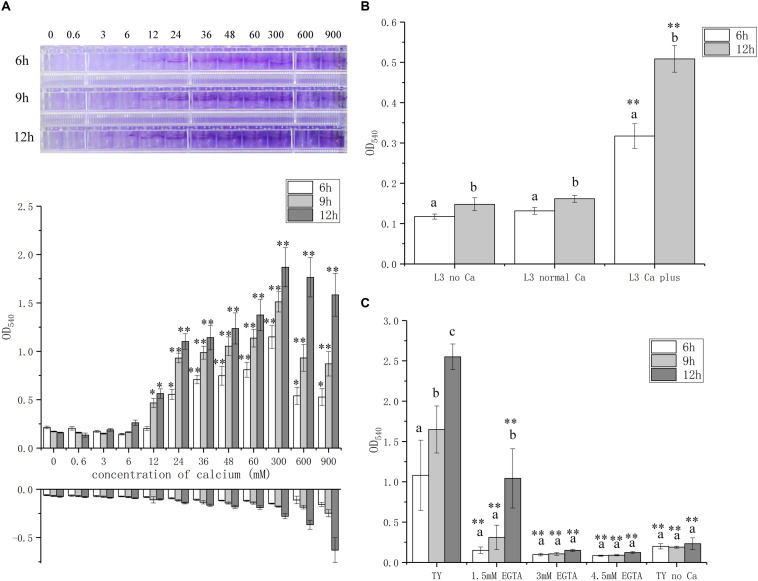
Biofilm formation of *A. caulinodans* with different concentrations of calcium and calcium chelator. **(A)** Calcium dose (0 to 900 mM) response of *A. caulinodans* biofilm formation with TY medium after 6, 9, and 12 h. Below the x axis means control without adding bacteria. Images on the top show the biofilm formed on the wall of 96-well plates. **(B)** Biofilm of *A. caulinodans* with different concentrations of calcium on L3 minimal medium after 6 and 12 h. **(C)** Biofilm for *A. caulinodans* with TY adding different concentration of calcium chelator, EGTA. Values are shown as the means and standard deviations from at least three independent experiments. The same letter above the error bars indicates not statistically different with results under same treatment by a Duncan’s test. Asterisks (^∗^) and (^∗∗^) mean *P* < 0.05 and *P* < 0.01 vs. the results after culturing same time with 0 mM calcium **(A)** or TY medium **(C)** by a Student’s *t* test.

To further explore the role of calcium on biofilm formation, we measured the biofilm biomass with various concentrations of calcium from 0 to 900 mM, which is lower or higher than the concentration of calcium, 6 mM, in standard TY medium. The biofilm biomass is positively correlated with the calcium concentration in the media and the highest level of biofilm formation was reached at 300 mM (Figure 2A). Though the biofilm biomass was high when 900 mM calcium was added, the control group without bacteria bacteria at this calcium concentration showed a false biomass increase up to OD_540_ of 0.6 (Figure 2A). In addition, the growth of *A. caulinodans* cells was inhibited when the concentration of calcium reached 60 mM calcium (Supplementary Figure S1). Therefore, calcium promotes biofilm formation, and high concentration of calcium impairs the growth of *A. caulinodans.*

Because TY medium is complex, other potential signal molecules may be present in tryptone or yeast extract that could confound the effect of calcium in biofilm formation. To exclude these possible confounding variables, we decided to test the effect of calcium on biofilm formation using L3 medium, containing sodium lactate and ammonium chloride as the sole carbon and nitrogen source, respectively. When 6 mM calcium was supplemented in the L3 medium, the biofilm formation increased from OD_540_ of 0.1 to 0.4 and 0.5 after 6 and 12 h, respectively (Figure 2B).

To further confirm the role of calcium in biofilm formation, various concentrations of Ca^2+^ chelator EGTA were added in TY medium. A weaker biofilm was formed when 1.5 mM EGTA was added compared to that without EGTA (Figure 2C). When 3 or 4.5 mM EGTA was added, there was no significant biofilm formation compared with TY medium without calcium (Figure 2C). These results strongly suggest that calcium is essential for the biofilm formation of *A. caulinodans*. To test whether the role of calcium on biofilm formation is common for divalent ion, we used Mg^2+^ to supplement into TY medium instead of Ca^2+^. In contrast to the strength of the effect of calcium on biofilm formation, Mg^2+^ did not promote, but inhibit, the biofilm formation of *A. caulinodans* (Supplementary Figure S3). This result indicates that the promoting role on *A. caulinodans* biofilm formation is exclusively calcium-dependent.

### Ca^2+^-Mediated *A. caulinodans* Biofilms Are Dependent on the Presence of eDNA

When we detected the biomass of biofilm under various concentrations of calcium with TY medium, we found that if the bacteria were washed with sterilized PBS before being used to form biofilm, a fairly higher concentration of calcium was required to trigger biofilm formation, in comparison with unwashed bacteria (Supplementary Figure S4). These results indicate the presence of extracellular matrix might be important for the promoting role of calcium on biofilm formation.

**FIGURE 3:**
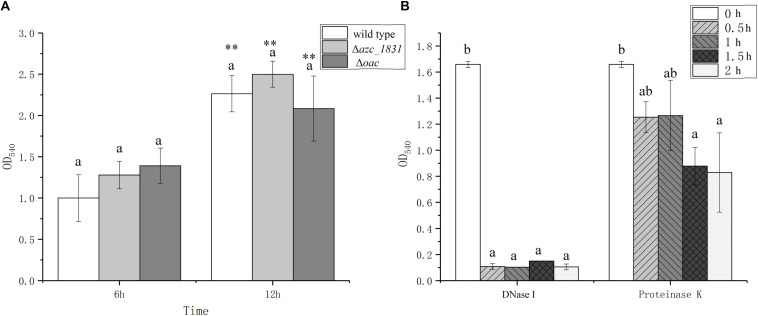
The roles of exopolysaccharides, eDNA, and extra cellular proteins on biofilm formation of *A. caulinodans*. **(A)** Biofilm formation of wild type, Δ*azc_1831* and Δ*oac* after 6 and 12 h with TY medium. **(B)** After 9 h, the biofilm biomass of wild type after adding DNase or Proteinase K from 0 to 2 h. Values are shown as the means and standard deviations from at least three independent experiments. The same letter above the error bars indicates not statistically different with results after culturing for same time **(A)** or same treatment **(B)** by a Turkey HSD’s test. Asterisks (^∗∗^) means *P* < 0.01 vs. the wild type after culturing for same time by a Student’s *t* test.

EPS is the main component of biofilm matrix for some bacteria (Tallawi et al., 2017). It has been reported that calcium promotes biofilm formation by inducing the expression of polysaccharide and cellulose synthesis genes (Tischler et al., 2018). In most cases, in the absence of exopolysaccharide synthesis and export, bacteria can adhere to surfaces but are unable to form multilayer biofilms (Karatan and Watnick, 2009). To study the role of EPS on the Ca^2+^-mediated biofilm formation, we measured the biofilm biomass of Δ*azc_1831* and Δ*oac* mutant strains, which delete one key gene or whole genes in cluster encoding EPS synthesis proteins (Sun et al., 2020). No matter at the 6 h or 12 h, however, there are no significant differences of biofilm biomass between Δ*oac* and wild type (Figure 3A).

In addition to EPS, protein and eDNA are two other important biofilm matrix in some bacterial species (Karygianni et al., 2020). When biofilm was formed with TY medium after 9 h, we added DNase or Proteinase K into the formed biofilm and tracked the changes of biofilm biomass after 0.5, 1, and 2 h. Addition of DNase can make the formed biofilm collapse quickly within 0.5 h, while adding Proteinase K only cause the biofilm biomass decrease slowly (Figure 3B). These results showed that the calcium-mediated biofilm of *A. caulinodans* is dependent on the presence of eDNA.

### Chemotaxis and Motility Are Required for Calcium-Mediated Biofilm Formation at Different Stages

**FIGURE 4:**
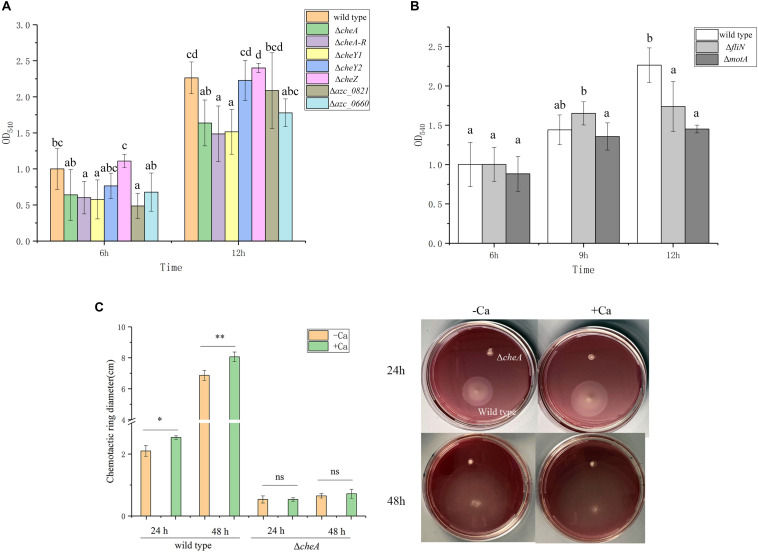
Biofilm formation of wild type and different chemotaxis and motility defective mutants. **(A)** Biofilm formation of chemotaxis defective mutants Δ*cheA*, *ΔcheA-R*, Δ*cheZ*, Δ*cheY1*, Δ*cheY2*, Δ*azc_0821*, and Δ*azc_0660* with TY medium after 6 and 12 h. **(B)** Two motility defective mutants Δ*fliN* and Δ*motA* form biofilm with TY medium after 6, 9, and 12 h. The same letter above the error bars indicates not statistically different with results under same culturing time by a Duncan’s test. **(C)** Swimming behavior of *A. caulinodans* wild type and Δ*cheA* on soft-agar plates with or without 6 mM calcium. Asterisks (^∗^*P* < 0.05; ^∗∗^*P* < 0.01) show a significant difference between conditions according to a *t*-test. Values are shown as the means and standard deviations from at least three independent experiments.

The relationship between chemotaxis and biofilm formation has been reported in many bacteria (Reinhardt and Bardy, 2018), and numerous studies suggest that transcription of chemotactic genes are activated during biofilm initiation (Karatan and Watnick, 2009). *A. caulinodans* has one core chemotactic pathway, including CheA, CheW, CheY1, CheZ, CheB, and CheR, which is similar to *Escherichia coli* (Sourjik and Wingreen, 2012), and 43 chemoreceptors. Additionally, *A. caulinodans* has a unique chemotaxis response regulator, CheY2, which functions as a phosphate sink (unsubmitted manuscript). To determine whether chemotaxis plays a role in *A. caulinodans* calcium-mediated biofilm formation, the biofilm biomass of different chemotaxis mutants including Δ*cheA*, Δ*cheA-R* (deletion of *cheA*, *cheY2*, *cheW*, *cheB*, and *cheR* operon), Δ*cheZ*, Δ*cheY1*, and Δ*cheY2* were measured after 6, 9, and 12 h with standard TY medium. Biofilm formation at 6 or 12 h, except Δ*cheZ* and Δ*cheY2*, the biomass of Δ*cheA*, Δ*cheA-R*, Δ*cheY1*, and Δ*cheY2* all showed significantly decrease compared to wild type (Figure 4A), indicating the chemotaxis plays an important role on these stages of biofilm. Two chemoreceptor mutants, Δ*azc_0821* and Δ*azc_0660*, were also employed to investigate the role of chemoreceptors on the calcium-mediated biofilm formation. The two chemoreceptors AZC_0821 (also termed as TlpH) and AZC_0660 (also termed as TlpA1) have broad-range ligands including organic acids and amino acids (Liu et al., 2017b; Liu et al., 2019b,c). At 6 or 12 h, they both showed a significant decrease of biofilm biomass compared to wild type (Figure 4A).

For many bacteria, the initiation of biofilm formation is dependent on flagellar motility (Jung et al., 2019). In addition to regulating swimming motility, the existence of flagellar filaments has been shown to help establish a robust biofilm (Hathroubi et al., 2018). To investigate the role of flagella and motility on the calcium-mediated biofilm, Δ*fliN* and Δ*motA*, two non-flagellated and non-motile mutants (Shen et al., 2018), were tested for biofilm formation. The biofilm biomass of both flagellar mutants was not significantly different from wild type at 6 h; however, the two mutants showed significant decrease of biofilm biomass after 12 h with standard TY medium (Figure 4B).

Considering the role of chemotaxis and motility on the calcium-mediated biofilm, we wonder what about the role of calcium on motility and chemotaxis of *A. caulinodans*? When we compared the swimming behavior of *A. caulinodans* in the presence and absence of 6 mM calcium using TY soft-agar plate assay. The result shows that calcium promotes the chemotactic motility of *A. caulinodans* significantly (Figure 4C). The non-chemotactic mutant Δ*cheA* was used as a control.

### Cyclic di-GMP and Quorum Sensing Proteins Are Involved in the Calcium-Mediated Biofilm Formation

**FIGURE 5:**
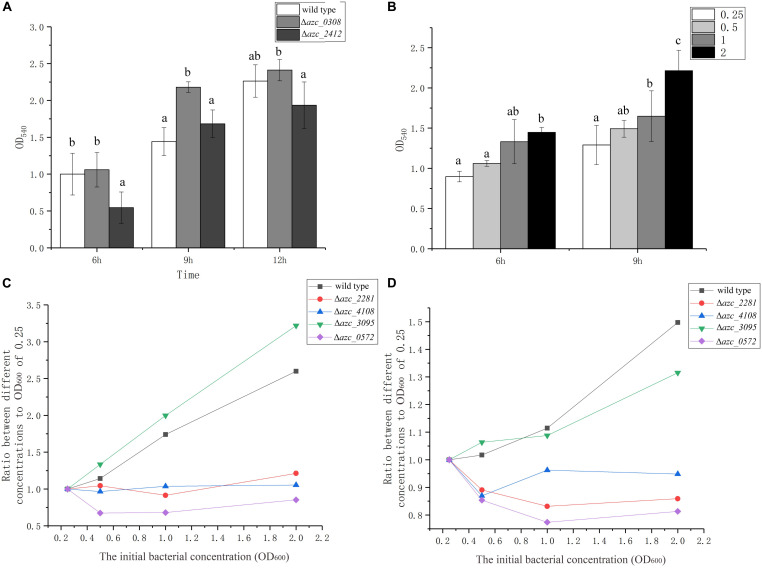
The role of c-di-GMP and quorum sensing on biofilm formation. **(A)** Biofilm formation of c-di-GMP increased and decreased mutants, Δ*azc_0308* and Δ*azc_2412* after 6, 9, and 12 h. **(B)** Biofilm formation of wild type with different initial bacterial concentrations from OD_600_ of 0.25 to 2.5 after 6 and 9 h. **(C,D)** The relationship between the biofilm biomass and initial bacterial concentration for different quorum sensing *luxR*-like gene mutants, Δ*azc_0572*, Δ*azc_2281*, Δ*azc_3095*, Δ*azc_4108* after 6 h **(C)** and 9 h **(D)**. Values are shown as the means and standard deviations from at least three independent experiments. The same letter above the error bars indicates not statistically different with results under same culturing time by a Duncan’s test.

Cyclic di-GMP is a conserved intracellular signal molecule that regulates motility, biofilm formation, and virulence in several species of bacteria (Zamorano-Sánchez et al., 2019). It has been reported that c-di-GMP can induce a calcium-binding protein CabA, which is essential for biofilm formation in *Vibrio vulnificus* (Park et al., 2015). In *A. caulinodans*, AZC_0308 (also termed as Chp1) is a c-di-GMP phosphodiesterase, and deletion of *azc_0308* causes an increase of intracellular c-di-GMP level (Sun et al., 2020). AZC_2412 is a diguanylate cyclase, and the intracellular level of c-di-GMP decreases when *azc_2412* is deleted (Yang et al., 2019). To investigate the role of c-di-GMP on calcium-mediated biofilm formation, Δ*azc_0308* and Δ*azc_2412* were tested for biofilm formation in standard TY medium. The Δ*azc_0308* showed an increase of biofilm biomass after 9 h compared to wild type, while Δ*azc_2412* showed a significant decrease of biofilm biomass compared to wild type after 6 or 12 h (Figure 5A). Interestingly, the biofilm biomass of wild type and Δ*azc_2412* were similar at 9 h (Figure 5A).

Quorum sensing can increase or decrease the intracellular levels of c-di-GMP level and regulate virulence and biofilm formation of bacteria (Karatan and Watnick, 2009; Papenfort and Bassler, 2016). Because the role of quorum sensing is dependent on the cell density, thus we determined the biofilm formation with different initial cell concentrations from OD_600_ of 0.25 to 2 after 6 or 9 h with standard TY medium. There is a positive correlation between the initial cell density of biofilm biomass of *A. caulinodans* (Figure 5B), indicating cell density may be involved in the calcium-mediated biofilm formation.

In gram-negative bacteria, LuxR-type receptors are employed to detect the autoinducers of quorum sensing (Papenfort and Bassler, 2016). Previously, we showed that one LuxR-type receptor AZC_0572 (also termed as AclR1) negatively regulates the biofilm formation of *A. caulinodans* with L3 minimal medium (Liu et al., 2019a). In addition to AZC_0572, there are eight other LuxR-type receptors in *A. caulinodans*, containing two functional domains of LuxR-type receptors, including a ligand-binding domain and a DNA-binding domain (Smith et al., 2006). In this study, the roles of four of them, AZC_0572, AZC_2281, AZC_3095, and AZC_4108, on the calcium-mediated biofilm were investigated. Except Δ*azc_3095*, the positive correlation between cell density and biofilm formation was disrupted in Δ*azc_0572*, Δ*azc_2281*, and Δ*azc_4108* (Figures 5C,D). This result further indicates the calcium-mediated biofilm is regulated by quorum sensing.

### The Role of Calcium on Colonization of *S. rostrata*

**FIGURE 6:**
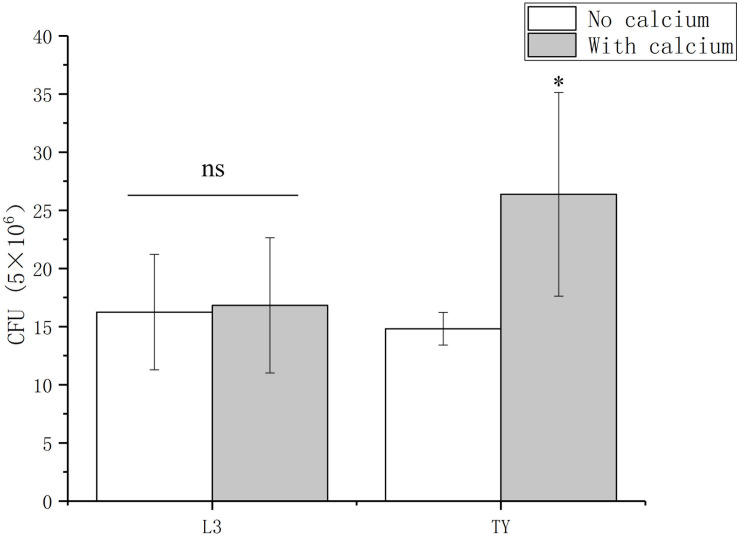
Colonization of wild type on *S. rostrata* roots with L3 or TY medium including calcium or not. The concentration of calcium in L3 and TY mediums is 0.36 and 6 mM, respectively. Values are shown as the means and standard deviations from at least three independent experiments. Asterisks (^∗^) means *p* < 0.05 vs. each medium without calcium by a Student’s *t* test.

For many bacteria, such as *Bacillus amyloliquefaciens*, *Enterococcus faecalis*, and *Vibrio fischeri*, the successful colonization of a host is influenced by their ability to attach and form a biofilm (Yaryura et al., 2008; Tischler et al., 2018; Xu et al., 2018; Willett et al., 2019). For *P. syringae* pv. Tomato DC3000, its association with host can be promoted by adding calcium (Fishman et al., 2018). These reports prompted us to determine the role of calcium on the *Sesbania* colonization of *A. caulinodans*. To test the role of calcium on the colonization of *A. caulinodans*, two kinds of mediums (L3 and TY) with or without calcium were used in a *S. rostrata* root colonization assay. In L3 medium, there is no significant differences on the colonization of *A. caulinodans* with or without calcium (0.36 or 0 mM) (Figure 6). In TY medium, the colonization of *A. caulinodans* was reduced significantly when calcium was removed from TY medium (Figure 6).

## Discussion

Biofilm formation is important for the establishment of rhizobacteria and legume plants associations (Scharf et al., 2016). The present work revealed that calcium plays a significant role in *A. caulindans* biofilm formation and *A. caulindans* colonization of *S. rostrata*. Also, we found that extracellular protein and eDNA are involved in the calcium-mediated biofilm formation. Additionally, we identified the effects of chemotaxis, motility, cyclic di-GMP, and quorum sensing on the calcium-mediated biofilm formation.

When yeast extract was removed from TY medium, *A. caulinodans* did not form biofilm, indicating the biofilm formation of *A. caulinodans* is correlated with the nutritional conditions. In addition to *A. caulinodans*, the biofilm formation of many other bacteria also depends on nutritional conditions. For example, *Bacillus subtilis* cannot form biofilm in mineral media (Voberkova et al., 2016), and nutrients are important for the biofilm formation of *Sinorhizobium meliloti* (Rinaudi et al., 2006).

When calcium was removed from or the calcium chelator, EGTA, was added into TY medium, both of them resulted in no biofilm formation, indicating that calcium is essential for the biofilm formation of *A. caulinodans*. Why calcium is essential for the biofilm formation of *A. caulinodans*? Calcium could promote the biofilm formation using several strategies. First, calcium can cross-link EPS. Some EPS in gram-negative bacteria have uronic acids, which contribute to the association with Ca^2+^ and enhance the mechanical stability of biofilm (Rinaudi et al., 2006; Flemming and Wingender, 2010). Second, calcium forms a bridge between eDNA. Calcium, as a divalent cation, could link different bacterial surfaces, which are negatively charged. It has been shown that eDNA at the cell surface can be bound to calcium and medicate bacterial aggregation in many bacteria such as *Haemophilus influenzae* (Das et al., 2017) and *Xylella fastidiosa* (Chen et al., 2017). Third, calcium can interact with biofilm-associated proteins. Biofilm-associated surface proteins are conserved in many bacteria, including *Pseudomonas putida*, *Salmonella enteritidis*, and *Pseudomonas fluorescens*, and they are important for the biofilm formation in early stage (Lasa and Penades, 2006). In *Salmonella*, biofilm-associated protein, BapA, is an important structural component for biofilm formation, and calcium was found to be important for proper protein folding (Guttula et al., 2019). In addition, biofilm-associated surface proteins are important for the maintenance of biofilm (McCall et al., 2019). Fourth, calcium can function as a signal and induce the expression of genes involved in different pathways to mediate biofilm formation. For example, in *Vibrio vulnificus*, in the presence of calcium, a LysR-type regulator, IamR, is induced and then affects pilus production and biofilm formation (Pu et al., 2020). In *Vibrio fischeri* and *Pseudomonas syringae* pv. tomato DC3000, calcium induces the biofilm formation and infection via two-component system (Cruz et al., 2012; Tischler et al., 2018). Calcium also can mediate the expression of *cabABC* operon, encoding a system to secret a calcium-binding matrix protein CabA, which is required for biofilm and rugose colony formation in *V. fischeri* (Park et al., 2015). In this study, exopolysaccharide mutants showed a similar phenotype as wild type on biofilm formation, indicating EPS does not influence the biofilm formation of *A. caulinodans*. However, another two main matrix components, eDNA and matrix proteins, especially eDNA, are involved in the biofilm formation and maintenance, because addition of DNase results in dispersal of biofilm. When we tested the biofilm biomass with different concentration with calcium from 0.6 to 900 mM, the maximum biofilm formation was observed with 300 mM calcium (Figure 2A), though the cells viability were decreased significantly at concentrations above 60 mM (Supplementary Figure S2). One possibility is that the effect of calcium on biofilm formation is modulated at least partially by cell death and lysis, releasing DNA into the medium. Then the enhanced amounts of eDNA might promote the calcium-mediated biofilm formation. Another possibility is that high osmolality caused by higher concentration of calcium may signal biofilm formation in *A. caulinodans*. These hypotheses need to be further studied via quantifying the amounts of eDNA of biofilm under different conditions.

Chemotaxis and flagellum-based motility are important for biofilm formation in many bacteria (Holscher et al., 2015). The result in this work further indicates that the calcium-mediated biofilm can be regulated by chemotaxis signaling in *A. caulinodans* in early and late stages. There is a positive correlation between aggregation and biofilm formation (Alexandre, 2015). We have shown that Δ*cheZ* is easier to form aggregates than wild type (Liu et al., 2018b). Consistent with previous results, there are significant differences between Δ*cheZ* and wild type or other chemotaxis mutants on the calcium-mediated biofilm formation. This result indicates that the role of CheZ on cellular processes other than chemotaxis might cause the phenotype of the mutant on biofilm formation. The Δ*cheY2* shows a similar phenotype as Δ*cheZ*, which functions as phosphate sink to terminate chemotactic signal transduction and might be involved in other cellular processes, such as aggregation. One possibility for the opposite roles of Δ*cheZ* and Δ*cheY2* on biofilm to that of the other mutants is the different swimming bias between them. Because lack of *cheZ* or *cheY2* will increase the clockwise rotation of flagella, while deletion of *cheY1* or *cheA* will increase the counter-clockwise rotation of flagella. The differences of flagellar rotation between them may influence the attachment of cells to plant root or 96-well surface.

There are two reasons for the negative role of two chemoreceptors AZC_0821 and AZC_0660 on biofilm formation. First, chemotaxis toward ligands in TY medium, which might be sensed by AZC_0821 and AZC_0660, is important for the calcium-mediated biofilm. In *Pseudomonas fluorescens*, the ligand citrate can bind to the Cache domain of GcbC to mediate biofilm formation (Giacalone et al., 2018). Second, calcium might function as a chemotaxis ligand, and AZC_0821 and AZC_0660 might be involved in the sensing of calcium signals directly.

Flagella-based motility is required for many bacteria in the initial stage of biofilm formation (Guttenplan and Kearns, 2013). However, flagella-based motility is not involved in the early stage of calcium-mediated biofilm formation in *A. caulinodans*. Interestingly, the absence of flagella decreases the biofilm biomass after 12 h. These results are consistent with the role of flagella in *Helicobacter pylori* biofilm formation, which plays a structural role in stabilizing biofilm (Hathroubi et al., 2018). According to some reports, calcium can enhance twitching motility in *Xylella fastidiosa* (Cruz et al., 2012, 2014), and promote swarming behavior of *Vibrio parahaemolyticus* (Gode-Potratz et al., 2010). Here, we further showed that calcium can also increase flagellar motility of *A. caulinodans.* Considering that FliN and MotA only play roles on the late stage of biofilm formation, these results indicate that flagellar motility is required for calcium-depended biofilm maintenance, but not initiation.

There are numerous studies about the role of c-di-GMP and quorum sensing on biofilm formation (Papenfort and Bassler, 2016). In this work, we found that increasing or decreasing the intracellular level of c-di-GMP, Δ*azc_0308* or Δ*azc_2412*, only affects the rate of biofilm development but not the maturation of biofilm. Because there are 37 GGDEF/EAL domain-containing proteins in *A. caulinodans*, and the role of *Δazc_0308* or *Δazc_2412* might be masked by other redundant proteins (Sun et al., 2019). Nine LuxR-like proteins were annotated in Microbe online website^1^. In this work, four of them were deleted separately, however, only three of them AZC_572, AZC_2281, and AZC_4108 showed a concentration-dependent role on biofilm formation. AZC_3095 might not play a role in the biofilm formation or its role is masked by other LuxR-like proteins. What needs to be pointed out is that, in this study, we only tested the biofilm formation of LuxR paralog mutants, and the direct role of quorum sensing on biofilm formation needs to be further studied.

The *A. caulinodans* colonization of *S. rostrata* is related to the attachment, biofilm formation and colonization. Calcium can promote the biofilm formation via bridging the negatively charged groups between the surfaces of plant and bacteria (Rinaudi et al., 2006). It is not surprising that the colonization of *A. caulinodans* can be enhanced. No differences of colonization using L3 medium with or without calcium indicate that there might be a concentration threshold of calcium to promote biofilm formation and colonization. In this study, however, we cannot exclude the possibility that the calcium in TY or L3 media might affect different plant physiology or growth of plant roots was limited within 4 h. In addition, the calcium concentrations vary with the soil types, and clayey soil has much higher calcium than sandy soil (Bonomelli et al., 2019), whether the promoting effect of calcium on the biofilm formation also applies to different soil types and rhizobacteria? These questions need to be answered using numerous studies in the future.

Taken together, these results from this work expand our horizon with regard to calcium in the biofilm formation and colonization of *A. caulinodans*. The mechanism of inducing biofilm through calcium remains unknown, and potential biofilm associated proteins, which are involved in the calcium sensing or binding, need to be further studied.

## Materials and Methods

### Strains and Media

*Azorhizobium caulinodans* ORS571 wild type, its derivative mutants, and all plasmids used in this work are listed in Table 1. Tryptone-yeast extract (TY) medium and L3 minimal medium and their modified medium were used to culture *A. caulinodans* wild type and its derivative mutants. Calcium chloride was used as the source of calcium ranged from 0.6 to 900 mM in TY medium and from 0.36 to 6 mM in L3 medium. *E. coli* and derivative strains were grown in Luria-Bertani medium.

**TABLE 1:** Bacteria strains and plasmids used in this study.

Strain or plasmid	Relevant characteristics^a^	Source or references
**Strains**		
***E. coli***		
DH5α	F- *supE44 AlacU169 (ϕ80 lacZΔM15) hsdR17 recA1 endA1 gyrA96 thi-1 relA1*	TransGen
*Azorhizobium caulinodans*		
ORS571	Type strain; Amp^R^, Nal^R^	Dreyfus et al., 1988
Δ*oac*	ORS571 derivative, deleting *oac* cluster, Amp^R^, Nal^R^, Gm^R^	Sun et al., 2020
Δ*azc_1831*	ORS571 derivative, deleting *azc_1831* gene, Amp^R^, Nal^R^, Gm^R^	Liu et al., 2019b
Δ*cheA*	ORS571 derivative, deleting *cheA* gene, Amp^R^, Nal^R^, Gm^R^	Liu et al., 2018a
Δ*cheA-R*	ORS571 derivative, deleting *che* cluter, including *cheA*, *cheY2*, *cheW*, *cheB*, and *cheR*, Amp^R^, Nal^R^, Gm^R^	Liu et al., 2018a
Δ*cheZ*	ORS571 derivative, deleting *cheZ* gene, Amp^R^, Nal^R^, Gm^R^	Liu et al., 2018b
Δ*cheY1*	ORS571 derivative, deleting *cheY1* gene, Amp^R^, Nal^R^, Gm^R^	Liu et al., 2018a
Δ*cheY2*	ORS571 derivative, deleting *cheY2* gene, Amp^R^, Nal^R^, Gm^R^	Liu et al., 2018a
Δ*azc_0821*	ORS571 derivative, deleting *azc_0821* gene, Amp^R^, Nal^R^, Gm^R^	Liu et al., 2019b
Δ*azc_0660*	ORS571 derivative, deleting *azc_0660* gene, Amp^R^, Nal^R^, Gm^R^	Liu et al., 2019c
Δ*motA*	ORS571 derivative, deleting *motA* gene, Amp^R^, Nal^R^, Gm^R^	This study
Δ*fliN*	ORS571 derivative, deleting *fliN* gene, Amp^R^, Nal^R^, Gm^R^	Shen et al., 2018
Δ*azc_0308*	ORS571 derivative, deleting *azc_0308* gene, Amp^R^, Nal^R^, Gm^R^	Sun et al., 2020
Δ*azc_2412*	ORS571 derivative, deleting *azc_2412* gene, Amp^R^, Nal^R^, Gm^R^	Yang et al., 2019
Δ*azc_3095*	ORS571 derivative, deleting *azc_3095* gene, Amp^R^, Nal^R^, Gm^R^	This study
Δ*azc_0572*	ORS571 derivative, deleting *azc_0572* gene, Amp^R^, Nal^R^, Gm^R^	Liu et al., 2019a
Δ*azc_2281*	ORS571 derivative, deleting *azc_2281* gene, Amp^R^, Nal^R^, Gm^R^	This study
Δ*azc_4108*	ORS571 derivative, deleting *azc_4108* gene, Amp^R^, Nal^R^, Gm^R^	This study
**Plasmids**		
pCM351	Allelic exchange vector, Gm^R^, Tc^R^	Marx and Lidstrom, 2002
pRK2013	Helper plasmid, ColE1 replicon; Tra+ Km^R^	Ditta et al., 1980

*^*a*^Amp^*R*^, ampicillin resistance; Gm^*R*^, gentamycin resistance; Km^*R*^, kanamycin resistance; Nal^*R*^, nalidixic acid; Tc^*R*^, tetracycline.*

### Molecular Methods and Strain Construction

To construct *motA* knock-out in this study, upstream and downstream fragments (around 500–800 bp) of the *motA* gene were amplified with cognate primers listed in Table 2. Two amplicons and allelic exchange vector, pCM351 (Marx and Lidstrom, 2002) were digested with cognate restriction enzymes and then they were purified and linked together with T4 DNA ligase. The resulting plasmid with upstream and downstream homology arms of *motA* was introduced into wild-type cells with the help of a helper pRK2013 (Ditta et al., 1980) using triparental conjugation. Correct mutant was selected using antibiotic gentamicin and verified by PCR and sequencing. For the construction of other mutants including Δ*azc_3095*, Δ*azc_2281*, and Δ*azc_4108*, the same method was used as above.

**TABLE 2:** PCR primers used in this study.

Primers	Sequences (5′-3′)^*a*^	Aim
MotA-UP-KpnI-F	GGTACCTCGCGGGTGTAGGCGACG	Construction of *motA* mutant
MotA-UP-NdeI-R	CATATGGCCCATGGCCATGAAGCC	Construction of *motA* mutant
MotA-DOWN-AgeI-F	ACCGGTAAGACCATTGCGGAAGGC	Construction of *motA* mutant
MotA-DOWN-SacI-R	GAGCTCACCGCGAAGTCCATGCGGGT	Construction of *motA* mutant
2281-UP-KpnI -F	GGGGTACCACATCGTCATCCATTCCG	Construction of *azc_2281* mutant
2281-UP-NdeI-R	GGAATTCCATATGCAACTGATGGATACCCG	Construction of *azc_2281* mutant
2281-DOWN-AgeI-F	GACCGGTATTCATTTTACGCATGATG	Construction of *azc_2281* mutant
2281-DOWN-SacI-R	CGAGCTCTCATCAGCGCCAGCCAGT	Construction of *azc_2281* mutant
3095-UP-KpnI-F	GGGGTACCTGAACGTCTGGTGGAATG	Construction of *azc_3095* mutant
3095-UP-NdeI-R	GGAATTCCATATGATGGCTGCGGTGCGCTAT	Construction of *azc_3095* mutant
3095-DOWN-AgeI-F	GACCGGTCACTGGCCCTGTCGGAA	Construction of *azc_3095* mutant
3095-DOWN-SacI-R	CGAGCTCCAACCATATGAGCTTCCT	Construction of *azc_3095* mutant
4108-UP-KpnI-F	GGGGTACCCGCCTGCTGCTCTCGGAA	Construction of *azc_4108* mutant
4108-UP-NdeI-R	GGAATTCCATATGTGCGCAGAGCCTCCAGAA	Construction of *azc_4108* mutant
4108-DOWN-AgeI-F	GACCGGTGCCCTGGCGAGCGTCTAG	Construction of *azc_4108* mutant
4108-DOWN-SacI-R	CGAGCTCGAACCTAACGCTCGACTC	Construction of *azc_4108* mutant

*^*a*^engineered restriction sites are underlined.*

### Biofilm Formation Assay

Glass tubes and 96-well plates were used to form biofilm. The initial concentration of cells was adjusted to OD_600_ of 2.5. Three milliliter and 200 microliter cell cultures were added into glass tubes and 96-well plates, respectively. Cells in glass tubes were incubated for 3–5 days at 37°C. For the cells in 96-well plates, cells were incubated from 0.5 to 12 h. After incubation, the glass tubes or 96-well plates were washed gently with sterile PBS and then adding 3 milliliters or 300 microliters 0.1% w/v Crystal violet in glass tubes or wells. The crystal violet was removed gently after incubating for 20 min at room temperature, and PBS was used to wash tubes or wells. Representative results of them were taken photos, and then 200 microliters of 30% acetic acid was added into each well. The OD_540_ of each well was determined by microplate reader (Tecan Infinite M200) after being transferred into a new 96-well plate.

### DNase and Proteinase K Assay

Biofilm was formed using method as above. After incubating for 9 h, the biofilm was used to determine the role of DNase and Proteinase K on the biofilm disperse. DNase I (50 μg/ml) and Proteinase K (200 μg/ml) were added into each well at 37° from 0.5 to 2 h, before being stained by crystal violet.

### Soft-Agar Plate Assay

*Azorhizobium caulinodans* wild type and derivative strains were cultured overnight with TY media. The overnight cultures were collected and washed with PBS at least two times. The cell suspension was then adjusted to an OD_600_ of 0.6 with PBS. Five microliter suspension were dropped into 0.3% soft-agar plates. The 0.3% soft-agar with or without 6 mM calcium (CaCl_2_) were poured 12 h before being used. The plates with cells were transferred to incubator at 37°. The diameter of chemotactic ring on each plate was recorded after culturing for 24 and 48 h.

### Colonization Assay

Sulfuric acid was used to sterilize the surface of *S. rostrata* seed, and then induced uniform germination. The sulfuric acid was removed after incubating 30 min, and sterile water was used to wash the seed at least five times. After washing, the seeds were immersed into sterile water and incubated in the dark condition for 48 h at 37°C. Overnight cultured cells were adjusted to OD_600_ of 0.01 using L3 medium or TY medium with or without calcium. Germinated seeds were soaked into cell suspension with different mediums for 4 h. Then, the surface of seedings was washed at least five times with sterile water. The washed seedlings were vortexed completely, and bacteria were reisolated from the surface of seedlings. After serial dilutions, 20 microliters of cells were plated on TY solid plates with antibiotics.

### Statistically Analysis

Differences among the treatments were statistically analyzed using the Statistical Package for the Social Sciences (version 20.0; SPSS Inc.). Student *t* test, Duncan test, and Turkey HSD test assuming equal variances (*P* < 0.05 or 0.01) were used to determine significant differences between treatments.

## Data Availability Statement

The raw data supporting the conclusions of this article will be made available by the authors, without undue reservation.

## Author Contributions

XL, KZ, and ZX conceived and designed the experiments, analyzed the data, prepared the figures and tables, and wrote the manuscript. XL, KZ, YL, DZ, and DW carried out the experiments. All authors approved the submitted manuscript for publication.

## Conflict of Interest

The authors declare that the research was conducted in the absence of any commercial or financial relationships that could be construed as a potential conflict of interest. The reviewer LM declared a shared affiliation with the authors to the handling editor at time of review.

## Footnotes

1 http://www.microbesonline.org/

## Supplementary Material

FIGURE S1 Growth curve of wild type with different modified TY and L3 medium. Values are shown as the means and standard deviations from at least three independent experiments.

FIGURE S2 Growth curve of wild type with different concentrations of calcium from 0 to 900 mM. Values are shown as the means and standard deviations from at least three independent experiments.

FIGURE S3 Biofilm biomass of wild type with different concentrations of magnesium instead of calcium after 6, 9, and 12 h. Values are shown as the means and standard deviations from at least three independent experiments.

FIGURE S4 Biofilm formation of wild type with different washing treatment. **(A)** Before testing the role of calcium on biofilm formation of *A. caulinodans*, bacterial cells cultured with TY medium were washed three times with TY no calcium medium. **(B)** Bacterial cells were cultured with TY medium without calcium, and then they were used to test the role of calcium on biofilm formation directly. Images at the top means the representative results of them. Values are shown as the means and standard deviations from at least three independent experiments.
