# Supplementary figures and images for: Effects of Calcium and Signal Sensing Systems on Azorhizobium caulinodans Biofilm Formation and Host Colonization
Source: Front Microbiol. 2020 Sep 16;11:563367. doi: 10.3389/fmicb.2020.563367 (PMC7533552; doi:10.3389/fmicb.2020.563367)

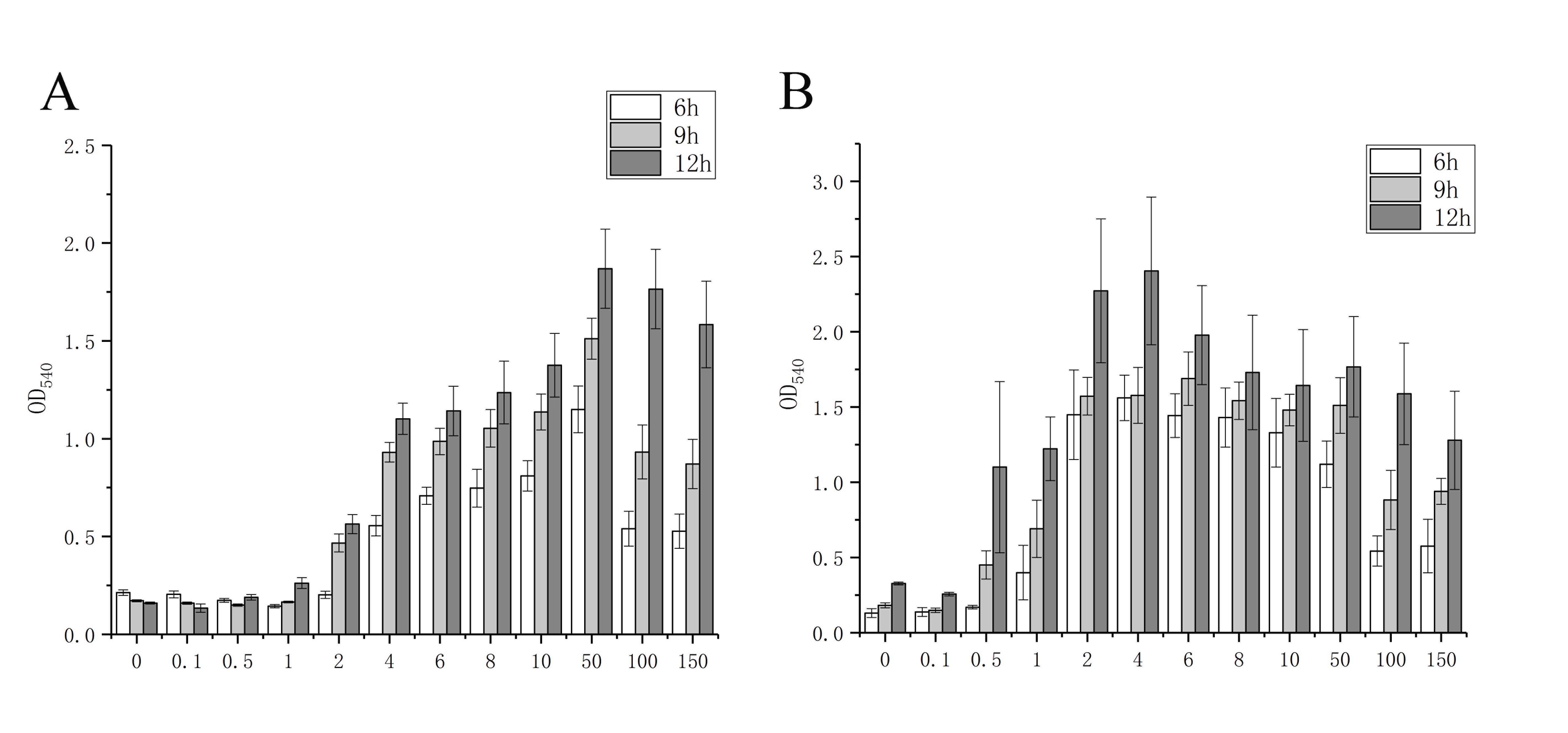

Supplement: FIGURE S4 — Biofilm formation of wild type with different washing treatment. (A) Before testing the role of calcium on biofilm formation of A. caulinodans, bacterial cells cultured with TY medium were washed three times with TY no calcium medium. (B) Bacterial cells were cultured with TY medium without calcium, and then they were used to test the role of calcium on biofilm formation directly. Images at the top means the representative results of them. Values are shown as the means and standard deviations from at least three independent experiments. [file Image_4.JPEG]
